# A Genetic Screen for Mutations Affecting Cell Division in the *Arabidopsis thaliana* Embryo Identifies Seven Loci Required for Cytokinesis

**DOI:** 10.1371/journal.pone.0146492

**Published:** 2016-01-08

**Authors:** C. Stewart Gillmor, Adrienne H. K. Roeder, Patrick Sieber, Chris Somerville, Wolfgang Lukowitz

**Affiliations:** 1 Department of Plant Biology, Carnegie Institution, Stanford, California, 94305, United States of America; 2 Department of Biological Sciences, Stanford University, Stanford, California, 94305, United States of America; Universidad Miguel Hernández de Elche, SPAIN

## Abstract

Cytokinesis in plants involves the formation of unique cellular structures such as the phragmoplast and the cell plate, both of which are required to divide the cell after nuclear division. In order to isolate genes that are involved in *de novo* cell wall formation, we performed a large-scale, microscope-based screen for Arabidopsis mutants that severely impair cytokinesis in the embryo. We recovered 35 mutations that form abnormally enlarged cells with multiple, often polyploid nuclei and incomplete cell walls. These mutants represent seven genes, four of which have previously been implicated in phragmoplast or cell plate function. Mutations in two loci show strongly reduced transmission through the haploid gametophytic generation. Molecular cloning of both corresponding genes reveals that one is represented by hypomorphic alleles of the kinesin-5 gene *RADIALLY SWOLLEN 7* (homologous to tobacco kinesin-related protein TKRP125), and that the other gene corresponds to the Arabidopsis *FUSED* ortholog *TWO-IN-ONE* (originally identified based on its function in pollen development). No mutations that completely abolish the formation of cross walls in diploid cells were found. Our results support the idea that cytokinesis in the diploid and haploid generations involve similar mechanisms.

## Introduction

Plants have adopted a unique mechanism of separating daughter cells in mitosis. This mode of cytokinesis involves two specialized organelles, the phragmoplast and the cell plate [1–3]. The phragmoplast is a microtubule based cytoskeletal array that, similar to the midbody of animal cells, forms from the remnants of the mitotic spindle. It is composed of two populations of anti-parallel microtubules that interdigitate with their plus ends at the division plane, or midline. Cytokinetic vesicles containing building material for the cross wall are transported along the phragmoplast microtubules and fuse at the midline to form a cell plate. The assembly of phragmoplast and cell plate typically grows from the center towards the periphery, where the cell plate fuses with the plasma membrane and existing wall (although polar growth has also been described [4]). The phragmoplast and the cell plate are defining traits of the plant lineage, but it remains largely unclear how and why this mechanism of cytokinesis has evolved. Its appearance seems to coincide with the evolution of plasmodesmata, direct connections between neighboring plant cells that are first established during cell division and are very likely a prerequisite for multi-cellular organization [5]. Detailed ultra-structural examinations of plant cytokinesis have been reported [6], but our knowledge of the underlying molecular machinery is still sparse.

Tobacco BY-2 cells, which can be synchronized with high efficiency, have been widely used to isolate plant proteins that might regulate mitosis. For example, the tobacco kinesin-related protein 125 (TKRP125) was purified from isolated BY-2 phragmoplasts by virtue of its ability to support gliding of microtubules *in vitro* [7]. TKRP125 is a member of the kinesin-5 family [8], which also includes vertebrate Eg5, fly KLP61F, *S*. *pombe* Cut7, and *E*. *nidulans* BimC (for kinesin nomenclature and phylogeny see [9]). In animal and yeast cells, kinesin-5 proteins form homo-tetrameric complexes that function as bipolar motors and organize the anti-parallel microtubules of the mitotic spindle (reviewed in [10,11]). Consistent with a role in cell division, TKRP125 co-localizes with the spindle and phragmoplast microtubules [8]. A weak allele of an Arabidopsis homolog of TKRP125, called *radially swollen7-1* (*rsw7-1*), was isolated on the basis of conditional root swelling [12,13].

Lateral growth of the phragmoplast and cell plate is dependent on the continuous removal of microtubules from the center and their re-assembly at the periphery, a process regulated by the NACK-PQR MAP kinase pathway (reviewed in [14]). Activity of the tobacco MAPKK kinase NPK1 peaks in late M phase, and dominant-negative variants block lateral expansion of the phragmoplast in BY-2 cultures [15]. Localization of NPK1 to the phragmoplast midline is mediated by the NPK1-activating kinesin-like protein 1 (NACK1) [15], a member of the kinesin-7 family [9]. The NPK1 MAP kinase cascade phosphorylates the microtubule-associated protein 65–1 (NtMAP65-1) [16], thereby down-regulating its bundling activity and promoting microtubule turnover.

Components of the NACK-PQR pathway have independently been identified through the analysis of Arabidopsis mutations impairing the formation of cross walls. Such mutations result in the development of enlarged cells containing multiple, polyploid nuclei and incomplete walls, suggesting that the phragmoplast or the cell plate are not fully functional. A number of genes mutating to this phenotype have been identified and can be placed into different functional categories. *HINKEL*, *PLEIADE*, and *RUNKEL* affect the organization of phragmoplast microtutules. *HINKEL (HIK*, also termed *AtNACK1*) encodes an Arabidopsis homolog of tobacco NACK1 [17], and *PLEIADE* (*PLE*, also called MAP65-3) encodes the Arabidopsis homolog of NtMAP65-1 [18,19]. RUNKEL (RUK) is a microtubule assocated kinase protein affecting microtubule stability and localization of HIK protein [20,21]. Three genes, the syntaxin *KNOLLE* (*KN*), the Sec1 homolog *KEULE* (*KEU*), and the predicted TRAPII tethering factor *CLUB* (also termed *TRS130*) affect membrane trafficking to the cell plate [22–25], a process that is tightly interwoven with phragmoplast organization. KN protein is only translated in mitosis and decorates cytokinetic vesicles as well as the growing rim of the cell plate [26], where it interacts with KEU protein to mediate the fusion of cytokinetic vesicles [24,27]. While *kn* and *keu* single mutants show incomplete cross walls and multinucleate cells in the embryo [22,23], *kn keu* double mutant zygotes completely lack the ability to form cross walls and, consequently, develop as a syncytium [28]. Gametophytic development is not affected in double mutants, implying that both *kn* and *keu* only affect cytokinesis in the diploid generation. Mutations in *CLUB* are associated with a slightly weaker phenotype and impair tethering of cytokinetic vesicles at the cell plate [29–31]. Two genes appear to affect the composition of the nascent cell wall: *ROOT-*, *SHOOT-*, *HYPOCOTYL-DEFECTIVE (RSH)*, encoding the hydroxyproline-rich protein EXTENSIN3 thought to act as a scaffold for wall assembly [32]; and *MASSUE*, encoding the callose synthase isoform GSL8 [33,34].

Mutations in the above genes were originally isolated in visual screens for aberrant seedling morphology. Such screens would have missed mutations that completely block the process. To circumvent this potential limitation, we searched for mutations disrupting cell division in the early Arabidopsis embryo, and recovered 35 mutations that result in *kn*-like phenotypes. These mutants comprised seven complementation groups, and included alleles of many of the genes mentioned above. In addition, we recovered mutations in two loci that are transmitted through the haploid gametophyte at a severely reduced rate. Map-based cloning revealed that they represent hypomorphic alleles of the Arabidopsis TKRP125 homolog *RSW7*; and of *TWO-IN-ONE* (*TIO*), the Arabidopsis ortholog of the FUSED serine/threonine kinase mediating hedgehog-dependent signaling in animals. Presumptive null alleles of *TIO* have been independently found based on their effect on cell division in pollen development [35]. No mutants were uncovered which, similar to *kn keu* double mutants, are completely deficient in cross wall formation.

## Materials and Methods

### Mutagenesis, plant growth, and tissue culture

Seed of the Landsberg *erecta* (L*er*) accession were mutagenized by imbibing in 0.3% ethylmethane sulfonate for 12 hours. From these seed, 25 batches of 500–1000 plants each were grown on soil (M1 generation), allowed to self fertilize, and bulk harvested in 25 pools of M2 seed. The M2 population was sampled relatively sparsely: 500–600 plants of each pool (~13,000 plants total) were first screened for individuals producing shrunken or collapsed seed upon self-fertilization; the embryos produced by ~7,000 selected plants were then analyzed as described below. Assuming each M1 plant contributed equally to the M2 pools, the rules of the Poisson distribution imply that ~40–60% of the M1 plants were represented in our M2 sample. However, M1 plants are genetic mosaics, with on average about 2 different sectors contributing to the M2 generation. Furthermore, embryo- or seedling-lethal recessive mutations, which were the target of our screen, are found in only 2/3 of the surviving M2 progeny (less, if transmission through the gametophytic generation is affected; see [36], for an in-depth discussion of these issues). While it thus seems likely that only a minority of all mutations present in the pools were examined, the sampling scheme benefited overall efficiency by keeping the number of duplicates, that is allelic mutations recovered from two or more plants of the same pool, low (3 cases among the 35 *kn*-like mutations described below). Mutations were considered independent if they originated from different pools or if they originated from the same pool but were in different complementation groups. Plants carrying reference alleles were crossed to wild type at least twice to reduce background mutations that might interfere with their analysis.

Plants were grown on commercial potting mix in walk-in growth chambers with continuous illumination at about 22°C and ~80 μmol/m^2^/s. Seedlings were grown on plates on 0.5X Murashige & Skoog basal medium (Sigma), supplemented with 1% sucrose, 1% agar, and 0.5 g/l MES (pH of 5.7), with continuous illumination of ~100 μmol/m^2^/s at 25°C or 16°C (permissive temperature for *rsw7-1*). Material for cell wall analysis was grown on media without sucrose.

### Histology and microscopy

For Nomarski microscopy, immature seed were dissected from the silique, embedded in Hoyer’s solution (7.5 g gum arabic, 100 g chloral hydrate, 5 ml glycerol, 30 ml water; for embryos that were at the early globular stage or younger, this mixture was diluted 2:1 with 10% gum arabic), allowed to clear for 4–12 hours, and examined with a Leica DMR microscope.

Confocal microscopy of embryos was performed as described [37]. Briefly, embryos were dissected from the seed, collected in 70% ethanol, extracted in 1:1 chloroform/methanol for 30 minutes and 100% methanol for 15 minutes, equilibrated in buffer (50 mM sodium phosphate, 0.05% Triton x-100, pH 7.2) and stained with Alexafluor 488 Hydrazide for two hours in the dark (Molecular Probes; 150 ug/ml in buffer). After rinsing, the samples were embedded in Hoyer’s solution and imaged with a Bio Rad MRC 1024 microscope (488 nm excitation, 520 nm emission).

For transmission electron microscopy, embryos were dissected from the seed coat and fixed in 4% formaldehyde, 0.25% glutaraldehyde. Embryos were postfixed with 1% osmium tetroxide, dehydrated through an ethanol series, transferred to acetone and infiltrated with Spurr’s resin (Ted Pella, Inc.) according to the manufacturer’s instructions. Ultra-thin sections were stained in 2% uranyl acetate and lead citrate and imaged using a Philips 4100 microscope.

Mature pollen was coated in a K550 sputter coater (Emitech Ltd.) and imaged with an S3500N scanning electron microscope (Hitachi), or stained with 1 μg/ml DAPI (5’,6 Diamidin-2-phenylindole; Sigma) and imaged with a Zeiss Axioplan 2 fluorescence microscope.

All images were processed and assembled using Photoshop and Illustrator software (Adobe Systems, Inc.).

### Analysis of cell wall composition

Material for cell wall analysis was extracted with 70% ethanol at 65°C, washed in acetone, dried and weighed. The neutral sugar composition of the non-cellulosic cell wall fraction was determined by gas chromatography of alditol acetates [38], with myo-inositol as an internal standard. Crystalline cellulose was measured colorimetrically with anthrone reagent [39,40].

### Molecular mapping

Mutants were mapped using PCR-based molecular markers in the F2 of a cross to the Columbia accession. Approximate map positions for *fackel*-like mutants were determined by bulked-segregant analysis [41]: three mutations map to the top of chromosome I, and one each to the bottom of chromosome I, the bottom of chromosome IV, and the top of chromosome V. *RUNKEL* (*RUK*) maps to chromosome III, between *CTR1* and *nga139* (30 and 16 recombinations in 210 meiotic events, respectively; both markers taken from the TAIR database; www.arabidopsis.org).

Markers used for fine-mapping *tio-10* and *rsw7-lph* are documented in Table 1. Informative recombinants were identified by *H029* and *H024* in the case of *tio-10*, and *L163* and *L653* in the case of *rsw7-lph*. The *rsw7-lph* mutation is linked in *cis* to the *erecta* mutation of L*er* (~4% recombination), and additional F2 plants harboring recombination events close to *rsw7-lph* were selected based on their *er* phenotype. Table 1 also lists a molecular marker that detects the *rsw7-lph* mutation and was used to identify heterozygous seedlings.

**Table 1:** PCR-based markers for molecular mapping.

Marker	Position	Primer pair	Comments
*mi208*	At1g49440	aggttacagttactaatgaag	Polymorphic EcoRV site
		atggaacgagataaacggagg	Col: ~260+90 bp; L*er*: ~360 bp
*H029*	At1g49750	gattggaggaggaaaaagtct	Length polymorphism (AT-repeat)
		tcacttatattgcattagacacc	Col: ~180 bp; L*er*<Col
*H033*	At1g49900/10	ccgtgaactcgacttatgcg	Polymorphic BfaI site
		ggtcgtccaactacaaatttcc	Col: ~380+330+110bp;
			Ler: ~480+330bp
*H803*	At1g49980	gttcatctgcccagaacagc	Polymorphic Bsp119I site
(C475803)		aggaacaaacacccctcagg	Col: ~300bp; L*er*: ~210+90bp
*OC1*	At1g50140	gcaccttttgcaacatatatcg	Polymorphic DdeI site:
		ctattgctggagcatcatcttc	Col: ~320+220bp;
			Ler: ~290+220+30bp
*H013*	At1g50150	ccctttcctatggacaatgtg	Length polymorphism (AT-repeat)
		aaatggcatgcattgtgaatcc	Col: ~160bp; L*er*<Col
*H375*	At1g50200	tagatgcagcaattatccccgc	Length polymorphism
(S375)		gttccgctattttcttctgagc	Col: ~210bp, L*er*: ~190bp
*H567*	At1g50230	catctgaggcaatgtgattgg	Polymorphic EcoRI site
(C425567)		ggttgtctctggattcacagc	Col: ~330+120bp; L*er*: ~450bp
*H560*	At1g50300	agatggtgactggatgtgcc	Polymorphic Bsh1236I site
(C425560)		tatccagcaacgctatatgcc	Col: ~470bp; L*er*: ~240+230bp
*H012*	At1g50330/40	gtcatgaaatcctaatacaggg	Length polymorphism (AT-repeat)
		aaaaacgtagtcatttcagaagc	Col: ~220bp; L*er*<Col
*H011*	At1g50410	aatatagatacgctctcatcgg	Length polymorphism (AT-repeat)
		tatggagtgagaattcacagtc	Col: ~240bp; L*er*>Col
*H024*	At1g50610/20	aaacccactttccctatcctg	Length polymorphism (CT-repeat)
		gagttttgcagcagctagaag	Col: ~180 bp; L*er*>Col
*H018*	At1g52110	ttggatgctcattacgttggc	Polymorphic SacI site
		atgagatgtcgtctgtcgtgg	Col: ~240+50bp; L*er*: ~290bp
*er*	At2g26330	tttggaaaatggtagcctctgg	dCAPS marker, creates
		ctaaaccttgtgctgcaccataAgct	polymorphic HindIII site
			Col: ~190bp; Ler: ~170+20bp
*L490*	At2g27190/200	ctcggaagagaaactcaactc	Length polymorphism
(C459490)		atctataaaccaataacccttcc	Col: ~280; L*er*: ~300bp
*L163*	At2g28150/60	ggagtatataaagtggatgaacc	Length polymorphism
(C451163)		tgaatagtgaagagtgaagacc	Col: ~250bp; L*er*: ~270bp
*L987*	At2g28400	cagtggtgaagaaacggtgg	Length polymorphism
(C458987)		aatttcgtagaaaacggtgacc	Col: ~220bp; L*er*: ~210bp
*L865*	At2g28510/520	atgattttcgctggattctgc	Length polymorphism
(C458653)		agggtcaatattggtacgagg	Col: ~190bp; L*er*: ~210bp
*L655*	At2g28570	ctctaagtgtctgtttgagtg	Length polymorphism
(C458655)		gtaataacattaactcattaagg	Col: ~190bp; L*er*: ~210bp
*L193*	At2g28690/700	atgaaacaatctgactttttccc	Length polymorphism
(C461192)		aatgttgtgtttacacttgcatc	Col: ~290bp; L*er*: ~320bp
*L302*	At2g28890	gaggttttggatggtcttagc	Length polymorphism
(C453302)		aaaactagtatctacgttccaag	Col: ~300bp; L*er*: ~280bp
*L653*	At2g28950/60	taatcccatctgtttctaaggg	Length polymorphism
(C449653)		tctttcgaagttttgatatccg	Col: ~330bp; L*er*: ~300bp
*LC15*	At2g28620	gtacctaaaacagtcaggaac	Polymorphic MboII site
(*rsw7-lph*)		ccaggcaatgaacagaggg	Wild type: ~390bp;
			rsw7-lph: ~330+60bp

Col and L*er* indicate the Columbia and Landsperg *erecta* accessions, respectively. The majority of markers were generated using publicly available collections of sequenced DNA polymorphisms (www.arabidopsis.org; the identifier numbers of these polymorphisms are listed below the marker designation, with “S” and “C” standing for the Stanford Genome Center and the Cereon/Monsanto Sequenced Nucleotide Polymorphism data set, respectively). PCR conditions: annealing at 60°C, 2mM Mg^++^ final concentration. Exceptions: the *H033* primer pair requires annealing at 58°C; furthermore, the *H033* PCR-product was ethanol precipitated prior to digestion, as BfaI is sensitive to Taq buffer. The *er* primer pair requires annealing at 58°C; this marker detects a point mutation inactivating the *ERECTA* gene of L*er*; a single nucleotide mismatch (capital “A” in the listing) was introduced in the sequence of the longer primer to generate a HindIII site in the PCR product derived from L*er* DNA. The *LC15* marker detects the *rsw7-lph* allele.

## Results

To better understand the molecular mechanisms of Arabidopsis cell division and, if possible, to obtain mutants completely blocked in cytokinesis, we conducted a genetic screen for mutant embryos segregating in self-fertilized siliques of heterozygous plants. Immature seed containing embryos between the globular and torpedo stage of development were optically cleared and examined by Nomarski microscopy. Approximately 7,000 plants producing ~25% shrunken or collapsed seed, a trait typical of all known cytokinesis-defective mutants, were selected for microscopy from an M2 population of ~13,000 ethylmethane sulfonate (EMS)-mutagenized Landsberg *erecta* (L*er*) plants (see Materials and Methods for details). In addition, we examined ~150 *emb* mutations induced by T-DNA transformation in the Wassilewskija accession and deposited in the Arabidopsis stock center (Columbus, OH), as well as ~50 X-ray-induced mutations resulting in shrunken seed (W. L., unpublished).

The majority of embryo-defective mutations caused morphological anomalies prior to the globular stage (see [42], for similar observations). Based on previous work, we expected that the loss of genes with a primary function in cell division would produce embryos consisting of fewer, abnormally large cells, containing two or more, typically polyploid nuclei. 70 independent mutations with this effect were recovered, representing <1% of the population (Table 2). In addition, very early arrest phenotypes, such as mutants consisting of a single cell, shaped like a large zygote, were observed in ~5% of the examined lines. However, these mutants typically had nuclei of normal size such that a primary defect in cell division seemed unlikely.

**Table 2:** Frequency of selected embryo-defective phenotypes.

Phenotypic class	Number recovered	Comments
**Fusca**	23 (~0.03%)	10 loci reported in [43].
**Radially swollen embryo**	25 (~0.04%)	5 loci reported in [37,44].
**Suspensor-less**	15 (~0.02%)	3 loci reported in [45].
**Cell division defective**	70 (~1%)	
** *titan/pilz*-like**	23*	5 loci reported in [46–48].
** *fackel*-like**	12	3 loci reported in [49–54].
** *knolle*-like**	35**	7 loci, see Table 3.

Only independent mutations are shown (see methods) and their approximate frequency in the population of ~7,000 embryo-defective mutations screened is listed in brackets.

* This number includes four mutations causing relatively weak phenotypes and representing hypomorphic alleles of *PFIFFERLING* and *KIESEL* (see text).

** This number includes the *kn* allele contained in line *4–43* (see text).

We assessed the saturation of our screen by estimating allelic frequencies for independent phenotypic classes. From the same sample, we isolated 23 independent *fusca* mutations (see [43], for a characterization of this phenotypic class), 25 independent mutations producing radially swollen embryos [37,44], and 15 independent mutations affecting the formation of a suspensor [45,55,56]. As inferred from the cumulative analysis of this collection, three to four mutant alleles per gene were recovered on average (Table 2). However, the frequency of mutant alleles does not appear to follow a normal distribution (discussed in [57]), such that many of the genetic loci remain represented by a single mutant allele (see below for examples). In some of these cases, transmission of the mutant allele through the gametophytic generation was reduced, while in others the mutant phenotype was comparatively subtle at the developmental stage we scored. We conclude that our screen was substantial enough to capture the genetic complexity of the target group with good accuracy, but did not reach statistical saturation.

### Phenotypic spectrum of cell division mutants

Most cell-division mutants can be readily assigned to one of three main phenotypic classes (Table 2). The largest class includes 34 mutants that are similar to *knolle* embryos [23], containing very large cells with multiple, highly polyploid nuclei. In contrast, the endosperm nuclei of these *kn*-like embryos appear of normal size.

The two other main classes of cell division mutants likely are caused by a primary defect that is distinct from cytokinesis. 19 mutants resemble *pilz*, *titan1*, and *titan5* embryos [46–48], consisting of only one or a few extremely large cells with extremely large nuclei. Similarly, their endosperm contains only one or a few nuclei, which often approach the size of a wild type embryo. The *PILZ/TITAN* genes encode tubulin folding factors [48,58], and their loss results in a rapid depletion of the free tubulin pool that eventually becomes insufficient to sustain the formation of mitotic microtubule arrays. 12 mutants resemble *fackel* embryos [49,50], characterized by an irregular, compact shape and a frequent occurrence of di-nucleate cells. *FACKEL* encodes a C-14 reductase in the sterol biosynthesis pathway, and mutations in two other genes of this pathway have been reported to result in similar embryonic phenotypes: *sterol methyltransferase1* / *cephalopod* and *hydra 1*, lacking a C-8,7 isomerase that acts downstream of *FACKEL* [51–54]. Sterols are an essential component of eukaryotic membranes, strongly impacting their physical and biological properties. However, it is as yet unclear how this defect relates to cell division. Neither the *pilz / titan* nor the *fackel* class was further analyzed.

**Fig 1:**
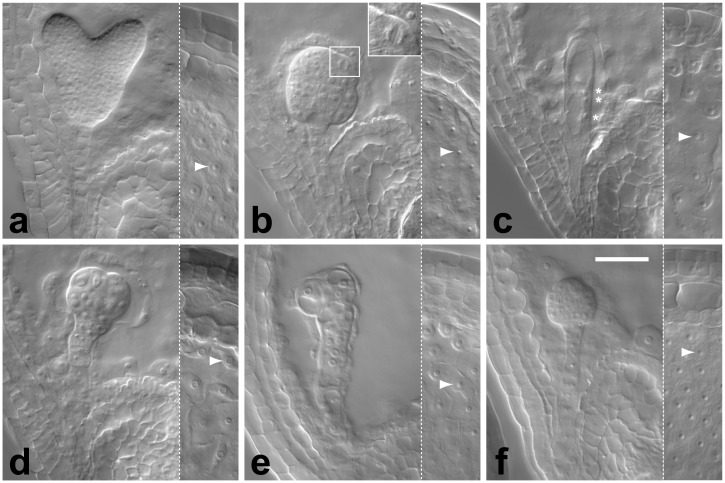
Range of cytokinesis-defective embryo phenotypes. Nomarski images of whole mount cleared immature seed; embryos at the heart stage of development are shown on the left and endosperm nuclei (arrowhead) on the right: (**a**) wild type; (**b**) *kn* mutant derived from line 4–43 (see text); enlarged cell caught in mitosis is boxed and shown magnified; (**c**) *kn keu* double mutant; three nuclei of normal size (stars) are visible; (**d**) phenotype produced by a weak *kiesel* allele; (**e**) double mutant phenotype produced by line 4–43; (**f**) mutant arrested at globular stage with no apparent cell division defects derived from line 4–43. Scale bar equals 50 μm.

The phenotypes observed in five lines seemed not fall into the main three classes described above, but rather resembled strong variants of *kn* mutants or weak variants of *kn keu* double mutants (Fig 1). However, a closer examination revealed that these lines also represent alleles of *PILZ / TITAN* genes and *KN*. Four of the five mutations also affect nuclear divisions in the endosperm, and typically fewer but somewhat larger endosperm nuclei were produced compared to wild type (Fig 1d). Mapping and complementation tests (not shown) demonstrated that these mutations represent hypomorphic alleles of the *PILZ*-group genes *PFIFFERLING* and *KIESEL* (the *kiesel* mutant is shown in the Fig 1; the four hypomorphic alleles were added to the total number of *titan / pilz*-class mutants in Table 2). The fifth line did not exhibit large endosperm nuclei (Fig 1e), but segregated a small fraction of embryos that were arrested in development without showing obvious cell-division defects. Subsequently, two mutations linked in *cis* (~15% recombination) could be separated in the progeny of this line: the first causes an arrest at the late globular stage (Fig 1f), while the second is an allele of *kn* (Fig 1b; this allele was added to the total number of *knolle*-class mutants in Table 2). Both mutations combined gave rise to the severe cytokineses-defective phenotype originally observed (Fig 1e). We conclude that our screen did not uncover a single mutation that completely or nearly completely abolishes cytokinesis in the Arabidopsis embryo, suggesting that such mutations are either rare or cannot be recovered.

### The *kn*-like mutations define seven genetic loci

A genetic analysis revealed that the 35 *kn*-like mutations represent seven genetic loci, five of which have been previously characterized (Table 3). Two alleles of *KN* [23], 15 alleles of *KEU* [22,24], nine alleles of *HIK / AtNACK1* [15,17], three alleles of *PLE* [18,19] and three alleles of *RUK* [21] were recovered.

**Table 3:** Loci mutating to kn-like phenotypes.

	Identifier	Protein	Mutations
***HIK* / *AtNACK1***	At1g18370	Kinesin-like [15,17]	8 EMS, 1 X-ray
***KEU***	At1g12360	Sec1 homolog [22]	14 EMS, 1 T-DNA
***KN* / *SYP111***	At1g08560	Syntaxin [23]	2 EMS
***RSW7***	At2g26620	Kinesin-like [13], this study	1 EMS
***TIO***	At1g50240	Fused-type protein kinase [35], this study	1 EMS, 1 X-ray
***PLE* / *MAP65-3***	At5g51600	MT associated protein [19]	3 EMS
***RUK***	At5g18700	MT-associated kinase-like protein [21]	3 EMS
	**Allele**	**Segregation**	**Germination**
***HIK* / *AtNACK1***	*OX5*	**19% (n = 896; p<0.005)**	>90%
	*20–55*	**19% (n = 498; p<0.005)**	n.d.
***KEU***	*AP77-18*	25% [8]	>90%
***KN* / *SYP111***	*AP6-16*	25% [8]	63% *
***RSW7***	*rsw7-lph*	**6% (n = 360; p<0.005)**	<5% *
***TIO***	*tio-10*	**17% (n = 1573; p<0.005)**	77%
	*tio-12*	**9% (n = 392; p<0.005)**	18% *
***PLE* / *MAP65-3***	*9–72*	26% (n = 625)	>90%
***RUK***	*7*	24% (n = 526)	85%

Gene identifiers and predicted or experimentally verified protein functions were taken from the TAIR database (www.arabidopsis.org). The number of EMS, X-ray, and T-DNA induced mutations isolated in this screen is listed in the “Mutations” column; the *keu* allele recovered from the Feldman collection of T-DNA insertion lines is not tagged with plasmid-derived sequences; the mutations used to determine segregation and germination rates are listed in italics. Segregation rates are expressed as fraction of mutant embryos in the progeny of self-fertilized heterozygous plants; “n” represents the total number of embryos analyzed; chi-square tests were performed to detect significant deviations from the expected Mendelian ratio, and the relevant p-values are included; where possible, two alleles were analyzed to confirm aberrant segregation. Germination rates were estimated by examining >500 progeny of self-fertilized heterozygous plants after incubation on MS plates for six days and are expressed as fraction of the mutants that had broken through the seed coat.

* A large portion of these seedlings (>1/3 for *kn*, and >1/2 for *lph* and *tio*) did not expand significantly beyond the size of the mature seed and remained white, suggesting that they had not survived dessication.

**Fig 2:**
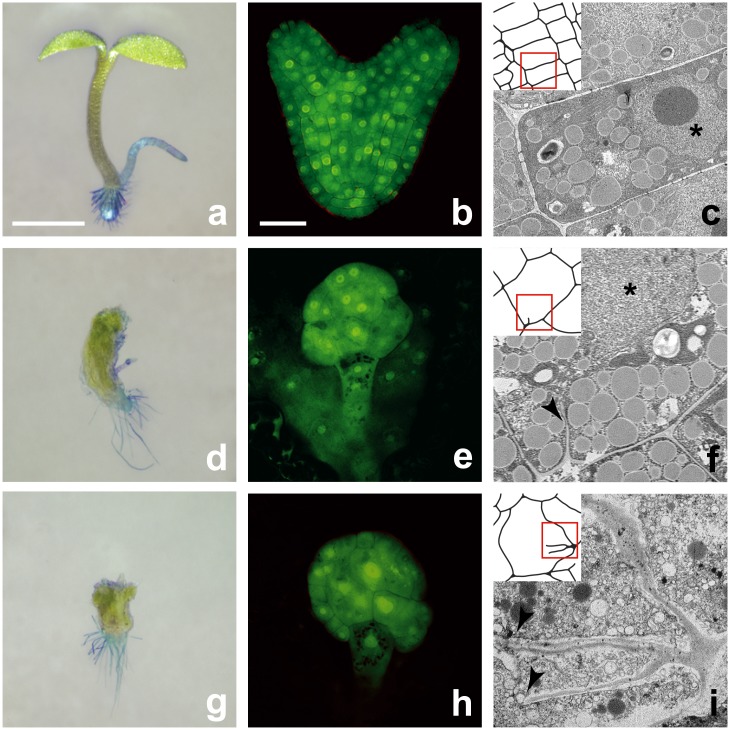
Morphological and ultra-structural characteristics of *tio* and *rsw* mutants. Left: Seedling morphology. Images of wild type (**a**) *tio-12* (**d**) and *rsw-lph* (**e**) seedlings; root hairs were contrasted with methylene blue; scale bar equals 1 mm. Center: Anatomy of embryos. Confocal micrographs of wild type (**b**), *tio-12* (**e**), and *rsw-lph* (**h**) embryos at the heart stage of development stained with Alexafluor 488 hydrazide; scale bar equals 30 μm. Right: Ultra-structure of embryonic cells. Transmission electron micrographs showing cells of wild type (**c**), *tio-10* (**f**) and *rsw-lph* (**i**) embryos; the arrowheads point to cell wall stubs, and the stars in (**c**) and (**f**) mark the nucleus; the side of panels equals 10 μm.

The remaining three mutations represented two loci tentatively named *OPEN HOUSE* (*OPN*; mutations *12–15* and *OX10*; [82]), and *LOOPHOLE* (*LPH*; mutation *15–150*). Molecular cloning (see below) revealed *opn* mutations are weak alleles of the fused type kinase encoding gene *TWO IN ONE* (*TIO)* which is required for gametophytic development [35] (we will refer to the alleles as *tio-12* and *tio-10*); the *lph* mutation is a strong allele in the kinesin-5 gene *RADIALLY SWOLLEN7* (*RSW7*) first identified on the basis of a weak conditional allele [12,13] (we will refer to this strong allele as *rsw7-lph*, to reflect the fact that we originally referred to this gene as *LOOPHOLE*). The *tio* and *rsw7* mutants we isolated result in perhaps even more pronounced morphological and ultra-structural defects than *kn*, *keu*, *hik* / *atnack1* or *ple* (Fig 2). Mutant seedlings are short and swollen compared to wild type, form no functional apical meristems and only rudimentary cotyledons, but develop relatively normal root hairs (Fig 2a, 2d and 2g). The epidermis consists of very large, bulging cells, resulting in a rough appearance. Cellular abnormalities indicative of cell division defects become apparent soon after fertilization (Fig 2b, 2e and 2h), and cell wall stubs or incomplete, gapped cell walls are frequent in the mutants (Fig 2f and 2i) when compared to wild type (Fig 2c).

Interestingly, *rsw-lph* as well as both *tio* mutations are poorly transmitted through the gametophytic generation (Table 3). This effect is most pronounced in *rsw7-lph*: self-fertilized heterozygous plants segregate only ~6% mutant progeny, as opposed to 25% expected for a Mendelian trait. The *tio* mutations we uncovered appear to be of different strength: while *tio-12* is associated with severe defects in the embryo, poor germination, and a strong gametophytic effect, *tio-10* appears to be generally weaker. A small but statistically significant gametophytic effect is also observed in *hik / atnack1* (19% instead of 25% mutants after self-fertilization, in two alleles tested), while *ple*, *ruk*, *kn*, and *keu*, as well as *kn keu* double mutants segregate in Mendelian ratios (Table 3; [28]).

### Seedlings of the *kn*-like class have a relatively normal cell wall composition

Incomplete cell walls and cell wall stubs can arise from defects in phragmoplast formation or membrane transport to the cell plate, but have also been found in mutants that affect cell wall biogenesis. For example, the walls of *korrigan* (*kor*) mutants appear generally disorganized when examined by electron microscopy and have an altered chemical composition, including relatively low cellulose content [59,60]. As a consequence, mutant seedlings are severely dwarfed, deficient in elongation, and often show gapped cell walls. KOR protein, which is predicted to function as an endoglucanase, shows a complex association with different membrane compartments but is found at the growing cell plate in mitosis [61–63]. It has been proposed that KOR might be required for the proper formation or processing of crystalline cellulose microfibrils. The wall stubs observed in mutants could arise from a failure of properly assembling the wall material of the cell plate. Alternatively, the cell walls of *kor* seedlings could be fragile and have a tendency of breaking.

We have investigated the possibility that a defect in cell wall biosynthesis contributes to the phenotype of *kn*-like mutants by comparing the chemical composition of wild type and mutant seedlings (S1 Table). The differentiation of secondary cell walls found in many mature cell types does not occur until after germination. To capture the range and dynamics of the associated changes in cell wall composition, three developmental stages of wild type were sampled: mature embryos, dissected from the seed coat at the late bent cotyledon stage, and seedlings harvested one or three days after germination (d.a.g.; germination defined as radicle emergence). Moreover, the shoots, hypocotyls and roots of seedlings harvested 3 d.a.g. were manually dissected and collected separately. We then determined the crystalline cellulose content of these samples as well as the neutral sugar composition of the non-cellulosic cell wall fraction. The results reveal substantial changes within the first three days of germination. Most notably, the overall cellulose content more than doubles. In addition, the walls of roots and shoots begin to display characteristic differences in their neutral sugar composition, with hypocotyls (as suggested by their anatomy), resembling an intermediate.

Mutant seedlings of the *kn*-like class typically germinated late and, thus, were harvested six days after their wild type siblings had germinated. Due to a pronounced gametophytic effect and extremely low germination, *rsw7-lph* seedlings could not be included in the study. Similar reasons made it difficult to collect the required amount of tissue in the case of *tio-12* mutants, so the weaker *tio-10* mutation was examined instead. In contrast to *kor* [59,60] and other embryo- or seedling-lethal mutants with an established effect on cell wall biogenesis, such as *cyt1*, *knf*, and *pnt* [64,37,44], the cellulose content of *kn*-like mutants is very similar to wild type seedlings harvested 3 d.a.g. An interpretation of the neutral sugar contents is more difficult, as they change substantially over the course of germination. However, the values obtained for mutants generally fall well within the range defined by the three tissue samples of wild type seedlings harvested at 3 d.a.g. (cotyledon, root, and hypocotyl). Deviations were only observed with arabinose (elevated by ~90% in *kn*), xylose (marginally reduced in *keu*), and galactose (reduced ~7–30% in *keu*, *kn*, and *ruk*). These difference may be related to the slow and, eventually, arrested development of the mutants. For example, the arabinose and galactose content of *kn* seedlings is more similar to wild type embryos than to wild type seedlings. Arabinogalactan-proteins [65], which likely contribute substantially to the measured pools of arabinose and galactose, are abundant in seed and accumulate in specific developmentally regulated patterns [66]. It seems possible that their turnover is delayed in *kn* seedlings. However, we do not expect that such differences impact the formation or stability of cross walls.

### Hypomorphic alleles of the Arabidopsis FUSED ortholog *TWO-IN-ONE (TIO)*

**Fig 3:**
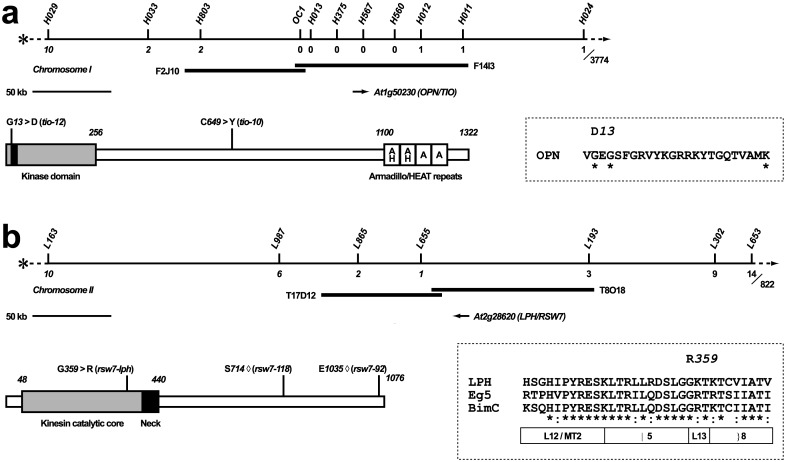
Molecular cloning of *OPN/TIO* and *LPH/RSW7*. (**a**) Top: Genetic and physical map of the *OPN/TIO* region on the lower arm of chromosome 1. Numbers below the line indicate the number of recombination events between *opn/tio* mutations and the corresponding molecular markers (with italics referring to recombinants on the centromeric side, and the total number of meiotic events analyzed listed on the far right); two BAC clones (black bars) spanning the *OPN*/*TIO* transcription unit (arrow) are shown below the map. Bottom: Domain structure of the TIO protein. The N-terminus consists of a *FU*-type kinase domain (gray bar, with the ATP binding pocket in black); the C-terminus contains four repeat motifs, the first two of which share significant similarity with Armadillo and HEAT repeats (white boxes labeled “AH”), while the second two show borderline similarity to Armadillo repeats (“A”; [35]); the *tio*-*12* allele (mutation *12–15*) harbors a glycine to aspartic acid substitution in the ATP-binding pocket (listed in the dashed box, invariant positions of the consensus sequence marked with a star); the PROSITE consensus (motif no. P00107) of this sequence signature is: [LIV]-G-{P}-G-{P}-[FYWMGSTNH]-[SGA]-{PW}-[LIVCAT]-{PD}-x-[GSTACLIVMFY]-x(5,18)-[LIVMFYWCSTAR]-[AIVP]-[LIVMFAGCKR]-K; the *tio-10* allele (mutation *OX10*) harbors a cystein to tyrosine substitution in a portion of OPN without significant similarity to known motifs. (**b**) Top: Genetic and physical map of the *LPH/RSW7* region on the lower arm of chromosome 2, organized as in (**a**). Bottom: Domain structure of the predicted RSW7 protein. The N-terminal catalytic core (grey bar) and the neck domain (black bar) show strong similarity to members of the kinesin-5 family; the insertion sites of the two T-DNA alleles, *rsw7-118* and *rsw7-92*, are marked with diamonds; the *rsw7*-*lph* allele harbors a glycine to arginine substitution in a conserved portion of the catalytic core; an alignment of this portion with human Eg5/KIF11 and *E*. *nidulans* BimC (GenBank accession nos. P52732, P17120) is shown in the dashed box, with stars representing invariant and colons conserved positions; folding of this domain is shown below the alignment and inferred from the crystal structure of rat brain kinesin [67] (L12/MT2: loop 12, microtubule binding domain 2; α5: alpha helix 5, L13: loop 13, β8: beta sheet 8).

We identified the mutations responsible for the *tio-10* and *tio-12* phenotype by standard positional cloning [41]. Despite a relatively large mapping population of >3500 meiotic events, we were not able to map the *tio* mutations to an interval smaller than 150 kb (Fig 3a, flanked by the markers *H803* and *H012*), suggesting a relatively low recombination frequency in the *tio* region of chromosome I. This interval contained At1g50230, a gene encoding the Arabidopsis ortholog of animal FUSED (FU)-type protein kinases. Although FU is a component of the Drosophila and mammalian Hedgehog signal transduction pathway, a recent report has revealed that Arabidopsis FU functions in mitosis [35]. Mutations in this gene were first identified based on their effect on the male gametophyte and named *two-in-one* (*tio*), because they result in the production of bi-nucleate pollen (mature wild type pollen contains three nuclei, the two condensed nuclei of the sperm cells, and the larger nucleus of the vegetative cell). TIO protein localizes to the phragmoplast, and reducing TIO function in seedlings by RNA interference results in multi-nucleate cells with incomplete walls.

Sequence analysis revealed that both mutations cause amino acid substitutions in the *TIO* coding sequence. Two functional domains have been tentatively identified in the TIO protein: a C-terminal FU-type protein kinase catalytic domain, and an N-terminal putative interaction domain containing HEAT/Armadillo repeats [35] (Fig 3a). The weaker *tio-10* mutation introduces an amino acid exchange in the center of the protein, a region that shows no striking similarity to other proteins (cysteine at position 649 is substituted with tyrosine; tgc to tac). The stronger *tio-12* mutation substitutes an aspartic acid for the wild type glycine at position 12 (ggt to gat), one of the most conserved positions within the ATP binding pocket (Fig 3a, dashed box). Both alleles are hypomorphic, as the presumptive null allele *tio-3*, which harbors a T-DNA insertion immediately downstream of the catalytic domain and does not produce detectable levels of transcripts, completely abolishes transmission through the male gametophyte [35].

### The *rsw7-lph* mutation disrupts a kinesin-5 gene

The *rsw7-lph* mutation was mapped to a ~100 kb region of chromosome II (flanked by the markers L655 and L193; Fig 3b). This interval contains one of the four Arabidopsis homologs of tobacco kinesin-5 TKRP125 (At2g28620 or AtKRP125c; the other Arabidopsis homologs are: At2g36200 or AtKRP125b; At2g37420 or AtKRP125a; and the slightly more distantly related At3g45850; see [68,69]). In animal and yeast cells, kinesin-5 proteins organize the anti-parallel microtubules of the mitotic spindle (reviewed in [10,11]). Consistent with an analogous function at the plant spindle or phragmoplast, At2g28620 transcription is up-regulated about two-fold in mitosis [70].

**Fig 4:**
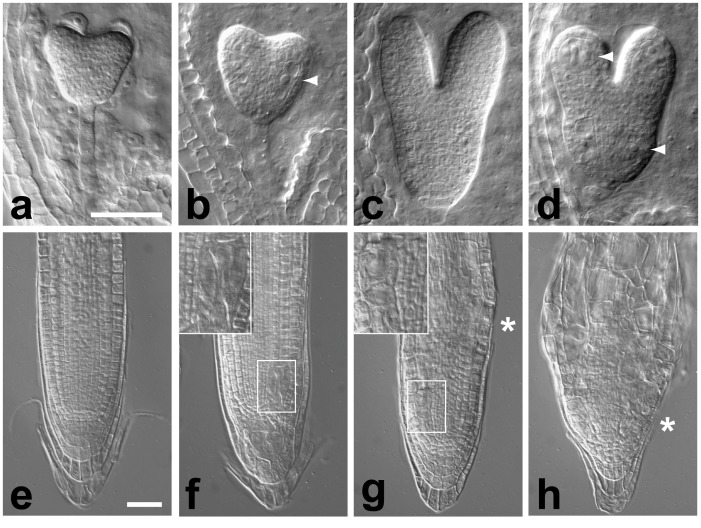
An allelic series of the *RSW7* gene. (**a–d**): *kn*-like phenotype of *rsw7-lph*/*rsw7-1* embryos. Nomarski images of whole mount cleared immature seed containing wild type (**a**,**c**) and trans-heterozygous embryos (**b**,**d**) at the early heart (**a**,**b**) and torpedo stage (**c**,**d**); arrows point to examples of enlarged cells with polyploid nuclei. (**e–h**): Aberrant divisions in the root meristem of *rsw7-1* and *rsw7-lph*/*rsw7-118* seedlings. Nomarski images of wild type (**e**), and *rsw7-lph*/*rsw7-118* trans-heterozygotes (**f**), grown at 25°C; and *rsw7-1* grown at 16°C (permissive temperature) (**g**), and 25°C (non-permissive temperature) (**h**); examples of abnormally large cell within the meristem, presumably resulting from a failure of mitosis, are boxed in (**g**,**h**) and shown magnified (top); stars indicate the boundary between meristem and elongation zone, as marked by the appearance of highly vacuolated rectangular cells. Scale bars equal 50 μm.

The *rsw7-lph* mutation introduces an amino acid exchange in a highly conserved portion of the kinesin catalytic core domain of At2g28620 (Fig 3b, dashed box; glycine at position 359 is substituted with arginine; gga to aga). Independent molecular cloning of the conditional mutation *radially swollen 7–1* (*rsw7-1*), which causes meristematic arrest and isotropic swelling of the root at a non-permissive temperature [12], revealed that it harbors an amino acid substitution in the same transcription unit [13]. Moreover, the two mutations do not complement each other: when *rsw7-1* pollen is used to fertilize *lph*/+ flowers, a large fraction of the resulting embryos exhibit weak cell division defects. Trans-heterozygous embryos appear normal until the mid globular stage but then frequently develop enlarged cells containing one or more polyploid nuclei (Fig 4b and 4d). Strikingly, the aberrant cells are often embedded in relatively normal looking tissue. This observation might imply that trans-heterozygous cells randomly fail to divide with a low rate but, once an error has occurred, become locked in a catastrophic cycle. We conclude that the *lph* and *rsw7* phenotypes, although different, are both caused by mutations in the kinesin-5 gene At2g28620, and thus we have referred to the *lph* mutation as *rsw7-lph*.

The *rsw7-lph* allele is predicted to produce a full-length protein. In an attempt to isolate a protein null in *RSW7*, we have searched the public collection of sequence tagged insertion libraries. Several insertions in At2g28620 have been documented, but all of them cluster outside of the kinesin catalytic core in the C-terminal tail of the predicted protein, where no striking similarities to other proteins can be identified. The phenotypes associated with two representative insertions were analyzed: SALK_092974, here named *rsw7-92*, an insertion after the glutamate at position 1035; and SALK_188756, here named *rsw7-118*, an insertion after the serine at position 714 (slightly upstream of the insertion site noted in the database; signal.salk.edu/cgi-bin/tdnaexpress; [71]). In both cases, homozygous plants were viable and had a normal appearance. Furthermore, the rate of root growth on agar plates was indistinguishable from Col wild type (assayed at 25°C; not shown). Surprisingly, these results indicate that the C-terminus of the At2g28620 kinesin-5 is not critically important for its function.

We next fertilized *rsw7-lph* flowers with *rsw7-92* and *rsw7-118* pollen and examined the resulting progeny. Trans-heterozygous embryos showed no visible defects. However, abnormally large, polyploid cells were frequently found embedded in the root apical meristem of *rsw7-lph*/*rsw7-118* seedlings (Fig 4f; 37 of 129 seedlings from the cross *rsw7-lph*/+ x *rsw7-118*). In some cases, this phenotype was accompanied by slow or stunted root growth. Similar abnormalities were prominent in root meristems of *rsw7-1* seedlings grown at a permissive temperature (Fig 4g, compare to Fig 4h, showing a root tip grown at a non-permissive temperature). Moreover, the border between elongation zone and apical meristem in these seedlings was much closer to the root tip than in wild type, and the overall growth rate markedly reduced (not shown). Trans-heterozygous *rsw7-lph*/*rsw7-92* seedlings appeared largely normal, with the exception that their distal root cap occasionally contained one or two abnormally large cells (5 in 69 seedlings derived from the cross *rsw7-lph*/+ x *rsw7-118*; not shown; not observed in seedlings from self-fertilized *rsw7-lph*/+ plants). Thus, the mutant alleles of *RSW7* can be ordered in a series of decreasing strength: *rsw7-lph* (closest to a null allele) > *rsw7-1* > *rsw7-118* > *rsw7-92* (nearly equivalent to wild type).

### The *rsw7-lph* mutation severely affects mitosis in the male gametophyte

We made use of the weak but consistent cell division defects shown by *rsw7-lph*/*rsw7-1* embryos to examine the genetic basis underlying the non-Mendelian segregation of *rsw7-lph* by reciprocal crossing. 44% (670 of 1517) of the embryos produced by fertilizing *rsw7-lph*/+ flowers with *rsw7-1* pollen contained abnormally large cells with multiple polyploid nuclei. Although this is a statistically significant deviation from the ratio of 50% that is expected for a Mendelian trait (p<<0.005), the effect is rather small and indicates that the *rsw7-lph* mutation does not severely impair viability and function of the female gametophyte. In contrast, only 9% (123 of 1969) of the embryos resulting from *rsw7-1* flowers crossed with pollen of *rsw7-lph*/+ plants showed a mutant phenotype, indicating that transmission of the *rsw7-lph* allele through the male gametophyte is extremely poor.

**Fig 5:**
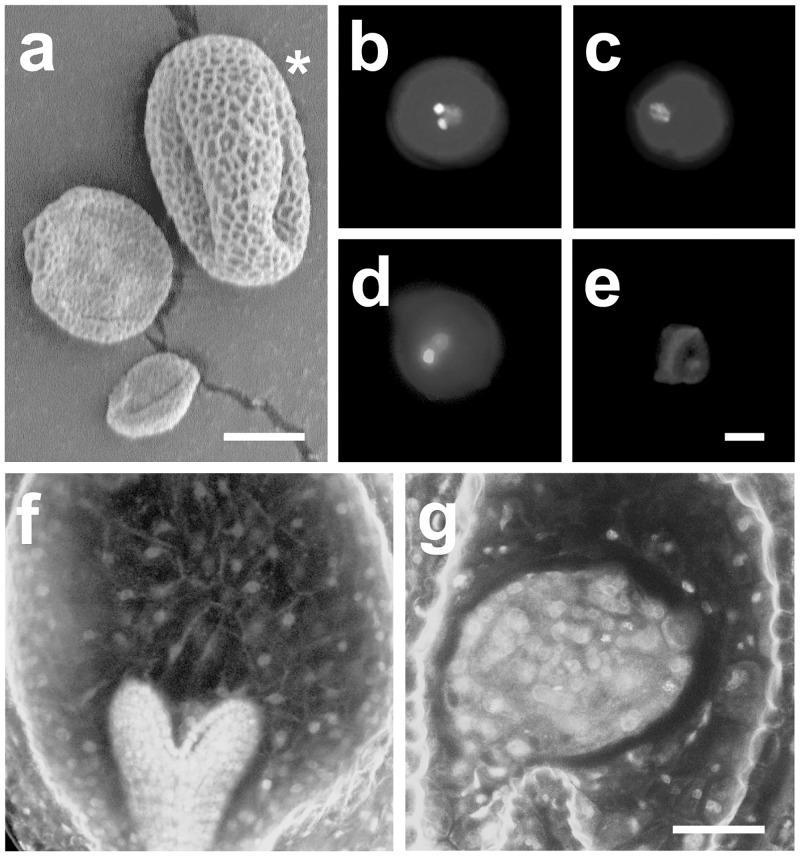
Effect of the *rsw7-lph* mutation on cellularization of the male gametophyte and endosperm. (**a**): Pollen produced by *rsw7-lph*/+ plants; scanning electron micrograph showing a mixture of normal (star) and shriveled or collapsed grains; scale bar equals 10 μm. (**b–e**): Cell division defects of *rsw-lph* male gametophytes. DAPI staining of normal pollen (**b**) reveals two small, brightly staining sperm cell nuclei and the large, less brightly staining nucleus of the vegetative cell; pollen of *rsw7-lph*/+ plants is frequently mono-nucleate (**c**), bi-nucleate (**d**), or collapsed with no detectable DNA (**e**); scale bar in (**e**) equals 10 μm. (**f, g**): Absence of a cellularized endosperm in *rsw-lph* seed. Confocal micrographs of Schiff-stained seed show the presence of cell walls in the endosperm of seed containing wild type embryos (**f**) but not in seed containing mutant embryos (**g**); scale bar in (**g**) equals 50 μm.

Nomarski microscopy revealed that close to 30% of the pollen grains produced by *rsw7-lph*/+ plants were small and shriveled (n>1000; compared to <5% in wild type, n>500; electron micrograph of pollen from *rsw-lph/+* shown in Fig 5a), suggesting their development was aborted. To explore to possibility that *rsw7-lph* impairs cell division in the male gametophyte, we stained mature pollen with the DNA-specific dye DAPI and examined the number and appearance of nuclei by fluorescent microscopy. Wild type pollen contains three nuclei: the two small and brightly staining nuclei of the sperm cells; and the larger, less intensely staining nucleus of the vegetative cell (Fig 5b). ~25% of the pollen grains from *rsw7-lph*/+ plants that were not shriveled contained an aberrant number of nuclei (n>600; compared to <5% in wild type, n>300). Most of these grains had a single, large nucleus that stained relatively weakly, resembling the nucleus of the vegetative cell in wild type (Fig 5c). Less frequent were grains with two large nuclei, one of which often stained more brightly (Fig 5d), or shriveled grains without any nuclei at all (Fig 5e). Taken together, it appears that only a minority of *rsw7-lph* pollen contains two sperm cells. Our examination of the endosperm in *rsw7-lph* seed also demonstrated a requirement for *RSW7* in cellularization of the endosperm. In wild type, cross walls between the endosperm nuclei have formed by the heart stage, while *rsw7-lph* seed of the same stage show a shrunken, cytoplasmically dense endosperm that remains syncitial (compare Fig 5f and 5g). These findings not only suggest an explanation for the low transmission of the *rsw7-lph* allele through the male gametophyte, but also confirm that *RSW7* is required for cytokinesis throughout the life cycle.

## Discussion

From a large-scale screen for Arabidopsis embryos with cytokinesis defects, we have recovered a phenotypically uniform class of >30 *kn*-like mutants, representing seven genes. Mutant embryos contain enlarged cells with multiple, often highly polyploid nuclei and incomplete walls, suggesting that cytokinesis is severely impaired but not completely blocked. Five of the seven genes, *KN*, *KEU*, *HIK* / *AtNACK1*, *RUK*, and *PLE*, are known to function at the phragmoplast or the cell plate (see introduction). Here we have identified hypomorphic mutations in two of the seven genes, *TIO* and *RSW7*, that were not previously known to cause a cytokinesis defect in the embryo.

While most *kn*-like mutations segregate 3:1 (wild type: mutant phenotype, as expected for recessive nuclear mutations) or with minimally distorted ratios, the mutant alleles of *tio* and *rsw7* are very poorly transmitted through the haploid generation. Molecular cloning revealed that these mutations are hypomorphic alleles of the Arabidopsis FU ortholog (*TIO*) and the kinesin-5 (*RSW7*). Presumptive null alleles of *TIO* have been independently isolated based on their effect on pollen development; they disrupt cytokinesis in the male gametophyte and impair cellularization of the female gametophyte, such that homozygous embryos are not obtained [35]. TIO protein localizes to the mitotic spindle as well as the phragmoplast midline, and reducing *TIO* expression in seedlings by means of inducible RNA interference blocks cytokinesis [35]. The hypomorphic alleles we isolated confirm a role for *TIO* in the cytokinesis of diploid cells. Furthermore, one of these hypomorphic alleles, *tio-12*, harbors a non-conservative substitution in a key residue of the catalytic domain, indicating that kinase activity is required for TIO function. The TIO orthlog FU in Drosophila binds Costal2, a member of the kinesin-4 family [9], through its catalytic domain as well as a C-terminal interaction domain [72,73]. A predicted interaction domain is present in the TIO C-terminus, but it shows relatively little sequence similarity to Drosophila FU. Despite this, interaction with a kinesin remains an attractive possibility, as it would explain the association of TIO with phragmoplast microtubules.

The Arabidopsis genome contains 61 kinesins, several of which associate with the phragmoplast in cell division (reviewed in [2]). Mutations in the kinesin-7 *HIK* / *AtNACK1*, which also localizes to the phragmoplast midline, result in *kn*-like embryos [17], making it a good candidate for a TIO binding partner. Another candidate might be the kinesin-5 RSW7. The strong *rsw7-lph* allele we recovered shows greatly reduced transmission through the gametophytic generation and overall very similar effects on pollen and embryo development as *tio* mutations. A weaker conditional allele of this gene, *rsw7-1*, has been isolated based on its temperature-sensitive root-swelling phenotype, which also indicates a defect in cytokinesis [12,13]. Notably, *rsw7-lph*, which harbors an amino acid exchange in the catalytic domain, is the only allele of *RSW7* from our screen. In contrast, we found nine alleles of the kinesin-7 *HIK*, demonstrating that kinesin-like genes make a large target for chemical mutagenesis. We propose that null alleles of *RSW7* likely are gametophytic lethal. In animal and yeast cells, members of the kinesin-5 family function as bipolar motors that organize the anti-parallel microtubules of the mitotic spindle (reviewed in [10,11]). It remains to be determined if *RSW7* plays an analogous role in the phragmoplast of plant cells.

Contrary to our expectation, we did not uncover mutations that mimic the phenotype of *kn keu* double mutants by completely or nearly completely blocking the formation of cross walls in the embryo. This negative result might in part be due to genetic redundancy, but gametophytic effects, as shown by *tio* and *rsw7* alleles, need to be considered as well. An implicit assumption of our approach was that cytokinesis in the diploid generation can be genetically separated from cytokinesis in meiosis and the haploid gametophytic generation. The Arabidopsis female gametophyte initially develops as a syncytium, and becomes cellular only after three rounds of nuclear divisions. In contrast, pollen development entails two complete mitotic divisions. Ultra-structural studies have suggested that cytokinesis in diploid cells differs significantly from cytokinesis in meiosis of pollen development and from cellularization of the female gametophyte (discussed in [74]). At first glance, this view appears consistent with genetic evidence: *kn keu* double mutations completely disrupt cell wall formation in diploid cells but have no detectable effect on the gametophytic generation [28]; conversely, *tetraspore* / *stud* (*tes*/*std*) mutations block cytokinesis during male meiosis but not in the diploid generation [75–76]. Thus, mutations with a specific effect on cytokinesis in the haploid or diploid generation can be found. However, it is not clear if these findings imply the existence of fundamentally different mechanisms. For example, post-meiotic cytokinesis in pollen is mediated by structures that closely resemble phragmoplasts and cell plates [77]. Furthermore, molecular cloning has revealed that *TES*/*STD* is identical to *AtNACK2*, the closest homolog of the *HIK*/*AtNACK1* kinesin-7 gene in the Arabidopsis genome [78,9]. Mutations in *HIK*/*AtNACK1* not only result in *kn*-like embryos [17], they are also transmitted through the gametophyte at a slightly but statistically significantly reduced rate (see above, Table 3). Both *NACK* kinesins are redundantly required for cellularization of the female gametophyte and postmeiotic cytokinesis in the male gametophyte [79,80]. In addition, *NACK* kinesins bind the NPK MAPKK kinase, enhancing its catalytic activity and mediating its transport to the phragmoplast midline [15]. Triple mutations that remove all three Arabidopsis orthologs of tobacco *NPK*, *the ANP* genes, cause both male and female gametophytic lethality, while *anp2 anp3* double mutant seedlings show cytokinesis defects with a low frequency [81]. Thus, the embryo-specific effect of *hik*/*atnack1* and the pollen-specific effect of *tet/std/atnack2* result from a recent gene duplication followed by the evolution of divergent transcriptional patterns, and do not reflect fundamental functional differences.

Instead, the available genetic evidence suggests that cellularization of the female gametophyte and cytokinesis in haploid and diploid cells might be mechanistically linked. Further extending this connection, *kn*, *hik*, *ruk*, *ple*, and *tio* alleles not only impair cytokinesis, but also cellularization of the free nuclear endosperm [82] (the *tio* alleles are referred to as *opn* in this study), a process also involving the formation of cell plate-like structures [83,84]. Our failure to isolate mutations that completely block cross wall formation in the diploid generation further supports the conclusion that, in all these contexts, separate cells might be generated by a common core machinery.

## Supporting Information

S1 Table Cell wall composition of kn-like mutants. For wild type (top section), six different tissues were measured: whole embryos dissected from the immature seed at the bent cotyledon stage; whole seedlings harvested one and three days after germination (d.a.g.); and manually dissected cotyledons, hypocotyls, and roots of seedlings harvested three d.a.g. Mutant seedlings (bottom section) were collected six days after their wild type siblings germinated, as their growth was significantly slower. Each sample contained ~100 seedlings. *: The amount of crystalline cellulose and neutral sugars of the non-cellulosic cell wall is expressed as a fraction of the total dry weight [μg per mg]; the relative abundance of individual neutral sugars was measured with respect to all six sugars analyzed [weight%]; all values represent the average of three measurements, with the standard deviation listed in brackets (exception: only a single measurement could be obtained for the neutral sugar content of embryos). ^†^: [64].
(PDF)
