# Supplementary material for: A Genetic Screen for Mutations Affecting Cell Division in the Arabidopsis thaliana Embryo Identifies Seven Loci Required for Cytokinesis
Source: PLoS One. 2016 Jan 8;11(1):e0146492. doi: 10.1371/journal.pone.0146492 (PMC4712874; doi:10.1371/journal.pone.0146492)
Supplement: S1 Table — For wild type (top section), six different tissues were measured: whole embryos dissected from the immature seed at the bent cotyledon stage; whole seedlings harvested one and three days after germination (d.a.g.); and manually dissected cotyledons, hypocotyls, and roots of seedlings harvested three d.a.g. Mutant seedlings (bottom section) were collected six days after their wild type siblings germinated, as their growth was significantly slower. Each sample contained ~100 seedlings. *: The amount of crystalline cellulose and neutral sugars of the non-cellulosic cell wall is expressed as a fraction of the total dry weight [μg per mg]; the relative abundance of individual neutral sugars was measured with respect to all six sugars analyzed [weight%]; all values represent the average of three measurements, with the standard deviation listed in brackets (exception: only a single measurement could be obtained for the neutral sugar content of embryos). †: [64]. (PDF) [file pone.0146492.s001.pdf]

Table 4: Cell wall composition of *kn*-like mutants.

	Wild type					
	**Embryos†**	**1 d.a.g.**	**3 d.a.g.**	**Cotyledon**	**Hypocotyl**	**Root**
**Dry weight** [ug]	2.6	7.7	11	7.1	1.7	2.1
**Cellulose***	31.0 (0.6)	69.5 (3.5)	105.5 (6.7)	n.d.	n.d.	n.d.
**Neutral sugars***	61	72 (8.7)	76 (3.6)	52 (5.5)	100 (2.0)	156 (7.1)
Rhamnose	5.2 (0.30)	10.1 (0.74)	11.2 (0.45)	15.8 (3.0)	11.8 (1.2)	6.8 (0.52)
Fucose	2.0 (0.16)	2.7 (0.06)	3.0 (0.05)	2.5 (0.21)	3.2 (0.28)	3.9 (0.51)
Arabinose	54.9 (1.1)	34.5 (1.7)	25.3 (1.5)	20.4 (1.8)	20.8 (0.91)	28.7 (1.2)
Xylose	19.5 (1.1)	17.1 (0.82)	19.5 (0.40)	23.6 (4.8)	23.1 (1.9)	19.3 (0.45)
Manose	4.8 (0.32)	4.7 (0.43)	4.3 (0.15)	6.9 (1.2)	5.3 (0.57)	3.0 (0.25)
Galactose	13.6 (1.7)	30.9 (0.99)	36.7 (2.0)	30.8 (1.1)	35.7 (0.18)	38.3 (1.4)

	kn-like mutants					
	hik	***keu***	***kn***	***opn***	***ple***	***ruk***
**Dry weight** [ug]	6	6.3	4.8	6	7.3	6.4
**Cellulose***	109.4 (9.5)	97.9 (4.2)	85.1 (12.7)	111.6 (9.3)	97.9 (23.4)	112.2 (21.8)
**Neutral sugars***	65 (13)	66 (2.3)	71 (5.0)	73 (9.0)	65 (2.2)	97 (9.7)
Rhamnose	10.5 (0.48)	9.7 (0.41)	10.2 (1.0)	11.4 (0.30)	14.8 (1.3)	11.1 (0.12)
Fucose	3.2 (0.49)	2.8 (0.06)	2.9 (0.31)	3.2 (0.14)	3.0 (0.17)	3.0 (0.12)
Arabinose	28.7 (2.4)	37.3 (0.63)	43.9 (1.8)	29.5 (0.36)	25.9 (1.3)	38.0 (0.73)
Xylose	21.4 (1.0)	16.8 (0.64)	18.0 (0.95)	21.7 (0.40)	20.6 (1.8)	19.3 (0.84)
Manose	5.0 (0.49)	4.8 (0.22)	3.5 (0.20)	5.0 (0.06)	4.9 (0.56)	3.4 (0.12)
Galactose	31.2 (1.7)	28.6 (0.51)	21.5 (1.1)	29.2 (0.40)	30.8 (0.69)	25.2 (0.12)
